# Real-time quantitative PCR with SYBR Green I detection for estimating copy numbers of nine drug resistance candidate genes in *Plasmodium falciparum*

**DOI:** 10.1186/1475-2875-5-1

**Published:** 2006-01-18

**Authors:** Isabel D Ferreira, Virgílio E do Rosário, Pedro VL Cravo

**Affiliations:** 1 Centro de Malária e Outras Doenças Tropicais/IHMT/UNL, Rua da Junqueira, 96, 1349-008, Lisbon, Portugal; 2 Centro de Malária e Outras Doenças Tropicais/UEI Biologia Molecular/IHMT/UNL, Rua da Junqueira, 96, 1349-008, Lisbon, Portugal

## Abstract

**Background:**

Evaluating copy numbers of given genes in *Plasmodium falciparum *parasites is of major importance for laboratory-based studies or epidemiological surveys. For instance, *pfmdr1 *gene amplification has been associated with resistance to quinine derivatives and several genes involved in anti-oxidant defence may play an important role in resistance to antimalarial drugs, although their potential involvement has been overlooked.

**Methods:**

The ^ΔΔ^Ct method of relative quantification using real-time quantitative PCR with SYBR Green I detection was adapted and optimized to estimate copy numbers of three genes previously indicated as putative candidates of resistance to quinolines and artemisinin derivatives: *pfmdr1*, *pfatp6 *(SERCA) and *pftctp*, and in six further genes involved in oxidative stress responses.

**Results:**

Using carefully designed specific RT-qPCR oligonucleotides, the methods were optimized for each gene and validated by the accurate measure of previously known number of copies of the *pfmdr1 *gene in the laboratory reference strains *P. falciparum *3D7 and Dd2. Subsequently, Standard Operating Procedures (SOPs) were developed to the remaining genes under study and successfully applied to DNA obtained from dried filter blood spots of field isolates of *P. falciparum *collected in São Tomé & Principe, West Africa.

**Conclusion:**

The SOPs reported here may be used as a high throughput tool to investigate the role of these drug resistance gene candidates in laboratory studies or large scale epidemiological surveys.

## Background

In the absence of a viable vaccine, current methods used for the control of malaria invariably rely on prevention through minimizing exposure to mosquitoes, and drug treatment of clinical disease. In areas of high transmission, where large numbers of malaria cases are presently inevitable, there is a reliance on anti-malarial drugs to treat the disease. However, parasite resistance, especially of *Plasmodium falciparum*, has been recorded to every anti-malarial drug currently in use [1].

It has been shown that differential copy numbers and/or differential transcription of putative drug resistance gene candidates may influence responses to drugs in malaria parasites. For instance, both in laboratory-adapted strains and clinical samples of *P. falciparum *increased copy numbers of the *pfmdr1 *gene have been implicated in resistance to quinine derivatives [2,3]. Additionally, changes in copy numbers of other genes, such as those coding for antioxidant defense enzymes, may be important in antimalarial drug resistance although these have not been fully explored as putative candidates [2,4]. In this context, is has been shown that higher levels of glutathione (GSH) in some parasites may help protect them from the toxic effects of chloroquine and thus contribute to resistance. Both in *P. falciparum *and in the rodent malaria parasite *Plasmodium berghei*, CQ-resistant lines contained higher levels of GSH than their sensitive counterparts [5], and this was related to increased expression of glutathione-s-transferase (*gst*) [6,7]. In addition, other anti-oxidant stress genes such as thioredoxins, have been widely shown to confer resistance to artesunate in tumor cells [8], but these have scarcely investigated in the context of drug-resistant malaria.

More recently, Uhlemann and colleagues provided strong evidence to indicate that resistance to artemisinins may depend on single nucleotide polymorphisms in the drug's putative chemotherapeutic target, the SERCA-type ATPase protein of *P. falciparum *(*Pf*ATP6) [9], although epidemiological evaluation of gene copy numbers in natural parasite populations has not been carried out.

Previous reports have described protocols for estimating copy numbers of the *pfmdr1*, using real-time quantitative PCR (RT-qPCR) [10,11]. Although the methods were shown to be highly sensitive, the fluorescent PCR signal was detected with TaqMan or hybridization probes specific for the *mdr1 *gene exclusively.

The objective of the present work was to develop protocols to estimate candidate gene copy numbers in genomic DNA extracted from dried blood spots of laboratory-adapted strains and field-collected isolates of *P. falciparum*, using RT-qPCR with SYBR green I detection. The genes included in the study were chosen on the basis of either a previous involvement in antimalarial drug resistance or their involvement in parasite responses to antioxidant stress (Table 1). SYBR Green I fluorescent dye has the important property of being a sequence-independent, universal RT-qPCR detection system, due to its ability to bind to all dsDNA molecules. Therefore, when working with a large panel of genes, using SYBR green instead of probes is another way to meet the demand of high throughput and to work more cost effectively.

**Table 1:** Genes analysed in this study

**Gene annotation**	**Abbreviation**	**Chromosome**	**Accession number**
actin (housekeeping)	β-*actin1*	12	NP_701803
multidrug resistance protein	*mdr1*	5	NP_703574
calcium-transporting ATPase (SERCA)	*atp6*	1	NP_703265
histamine-releasing factor, putative	*tctp*	5	NP_703454
glutathione peroxidase	*gpx*	12	NP_701484
glutathione reductase	*gr*	14	NP_702080
glutathione S-transferase	*gst*	14	NP_702075
fe-superoxide dismutase	*sod1*	8	NP_704405
trx peroxidase (2-Cys peroxiredoxin)	*trx1*	14	NP_702257
thioredoxin peroxidase	*trx2*	12	NP_701510

Although SYBR Green I detection is prone to lack of specificity, its comparative low price and ability to detect any given PCR product in a sequence-independent manner outweigh its potential disadvantages provided optimal conditions are assured.

## Methods

### DNA extraction from laboratory clones and field isolates of *P. falciparum*

A set of eight DNA samples of *P. falciparum *from the Democratic Republic of Sao Tomé & Principe (DRSTP) were used in this study. Blood samples had been collected previously by Passive Case Detection (PCD) as part of an on-going collaboration between Portugal and the DRSTP during the month of February 2004, from suspected malaria carriers attending the Centro Policlínico de Saúde de Água Grande, in the city of São Tomé.

No age restrictions were applied. After confirmation of *P. falciparum *infection by microscopical observation of thin and thick Giemsa-stained blood films, 1 ml of venous blood was collected into Monovettes containing EDTA, after individual informed consent and local ethical approval. A sub-sample of this was spotted onto Whatman n°4 filter paper and then parasite genomic DNA was obtained from all samples by boiling in Chelex-100 [12] followed by ethanol precipitation. A similar protocol was used to extract genomic DNA from the references strains *P. falciparum *3D7 and Dd2, which were kept in deep frozen stabilates and cultured *in vitro *at the time of these experiments.

### Oligonucleotide primer design

Using data deposited in *P. falciparum *GeneDB [13], *P. falciparum *3D7 individual cDNA gene sequences were retrieved and used as template for designing all real-time PCR oligos (Table 2).

**Table 2:** Real-time quantitative PCR primers

**Gene**	**Primer**	**Sequence (5' ⇒ 3')**	**Concentration (nm)**	**Amplicon size (bp)**
*Pf-β-actin1*	sense	GGA CAC ATA TTG TGC CTG C	300	90
	antisense	CTC CAC TAT CTA ACA CAA TAC C	300	
*Pf-mdr1*	sense	CAA GTG AGT TCA GGA ATT GGT AC	300	230
	antisense	GCC TCT TCT ATA ATG GAC ATG G	300	
*Pf-atp6*	sense	GCT GCA TTC ATT AGT TTC GTG	300	126
	antisense	GCC ATA CAC CTA CGG CAG C	600	
*Pf-tctp*	sense	CAA ATG ATG AAG TAT GTT CCG	300	55
	antisense	GGT ACT TCA AAT GGA TCT TGT TG	600	
*Pf-gpx*	sense	CGT CGA TAA AAA TGG AGA AGT TG	300	56
	antisense	CTA ACG GGT TTG TTT TGG GTG	600	
*Pf-gr*	sense	GCA GTG GCC TTA AAA ATG AAT G	300	68
	antisense	GCT GTA GGA TGT ATA GGT ATG G	300	
*Pf-gst*	sense	GAT GCA AGG GGT AAA GCT G	300	150
	antisense	GGG TAC TTG CTC AAA AGG AG	600	
*Pf-sod1*	sense	GAT TAC AGA AAT GAC AGA GCA TC	300	56
	antisense	CAA TTT ACT AGG TTC CAC CAA G	600	
*Pf-trx1*	sense	CAT ATG TAG GAA GAG AAG CTC C	300	53
	antisense	ATC TGC AAA AAC TGC TTC AGC C	600	
*Pf-trx2*	sense	CGC TAG TGA CAA AGA AGG C	300	60
	antisense	ACA AAC AAC AGT ATT TCT GAC C	600	

Real-time PCR oligonucleotide primers were manually designed for each of the genes to assure maximal efficiency and sensitivity, according to the following parameters: avoidance of the formation of self and hetero-dimers, hairpins and self-complementarity, primer length and melting temperature. These properties were verified using two different internet-based interfaces: Primer-3 [14] and Oligonucleotide Properties Calculator [15]. When possible, "GC clamps" were placed at the 3'-end of each primer to minimize breathing between primer and template DNA, which can promote mispriming and decrease efficiency. Primers were designed such that amplicon sizes ranged between 50 and 250 bps. Melting curve analysis was always performed at the end of each PCR assay to control for specificity; specific reactions should result in a single melting peak corresponding to the PCR product being amplified. In contrast, multiple melting peaks imply that that the reaction is either unspecific in that it originates more than one amplicon or that primer dimers are being formed. Either condition alters the validity of PCR kinetic parameters and, thus, any primer pairs producing more than one melting peak were discarded.

### Real-time PCR assays

Real-time PCR was performed in the GenAmp 5700 SDS^® ^(Applied Biosystems™), using the default thermocycler program for all genes: 10 minutes of pre-incubation at 95°C followed by 40 cycles for 15 seconds at 95°C and one minute at 60°C.

Individual real-time PCR reactions were carried out in 20 μl volumes in a 96-well plate (Applied Biosystems™) containing 1× buffer (10×), 3.5 mM MgCl_2_, 200 μM dNTPs, different concentrations of sense and antisense primers (Table 2), 0.025 U/μl enzyme and 1:66000 SYBR GreenI ^®^. All reactions were made using qPCR™ Core Kit for SYBR Green I^® ^(EUROGENTEC™).

At the end of each reaction, Cycle threshold (Ct) was manually setup at the level that reflected the best kinetic PCR parameters, and melting curves were acquired and analysed.

### Data analysis using the 2^-ΔΔCt ^method and its validation

The two most commonly used methods to analyse data from real-time, quantitative PCR experiments are absolute quantification and relative quantification. Absolute quantification is usually applied to determine the input copy number, usually by relating the PCR signal to a standard curve. Relative quantification is more commonly used to measure gene expression by relating the PCR signal of the target transcript in a treatment group to that of a sample such as an untreated control. Absolute quantification is usually more accurate but requires elaboration of standard curves for each gene under study that need be ran at each experiment, thus increasing experimental costs and significantly decreasing throughput.

In this work, the 2^-ΔΔCt ^method of relative quantification (described in detail in [16]) was adapted to estimate copy numbers in *P. falciparum *genes. This method allows to estimate gene copy numbers in unknown samples, requiring two main pre-requisites. The first is the existence of at least one calibrator consisting of template DNA with known copies of each of the studied genes. The second is the need to have a house-keeping gene of constant copy number in all samples, which permits normalization of the quantitative data. In this work, genomic DNA extracted from *P. falciparum *3D7, known to harbour a single copy of each gene studied, was used as calibrator, while *Pf*-β-*actin1 *served as the house-keeping gene in all experiments.

The ΔΔCt calculation for the relative quantification of target was used as follows ^ΔΔ^Ct = (Ct, target gene – Ct, *Pf*-β-*actin1*)χ – (Ct, target gene – Ct, *Pf*-β-*actin1*)_y_, where χ = unknown sample and y = *P. falciparum *3D7. After validation of the method, results for each sample were expressed in N-fold changes in χ target gene copies, normalized to *Pf*-β-*actin-1 *relative to the copy number of the target gene in *P. falciparum *3D7, according to the following equation: amount of target = 2^-ΔΔCt ^[16]. A minimum of two experiments was carried out for each gene and sample. At each experiment, each individual sample was run in triplicate wells and the Ct of each well was recorded at the end of the reaction. The average and standard deviation (SD) of the three Cts was calculated and the average value was accepted if the SD was lower than 0.38 [17]. Results for each sample were expressed as the N-fold copy number of a given gene relative to *P. falciparum *3D7, by calculating the geometric mean between the two experiments. Assays were repeated if amplification curves did not reflect exponential kinetic parameters or if the N-fold copy number of a given gene was lower than 0.7 or higher than 1.3. In cases where N-fold was comprehended between those values (0.7 <N-fold< 1.3), it was accepted that the test sample harboured a single copy of the target gene, i.e., N-fold = 1.

### Determination of real-time PCR efficiencies

For the ΔΔCt calculation to be valid, two important parameters must be considered beforehand. First, the efficiency of a given PCR amplification must be close to 100%, and second, the relative efficiency must be optimal, that is, the amplification efficiencies of the target and reference genes must be approximately equal [16]. In order to determine PCR efficiencies for each gene, *P. falciparum *3D7 genomic DNA was diluted in serial 10-fold ranges and the Ct value at each dilution was measured. A curve was then constructed for each gene from which efficiency was determined. Real-time PCR efficiencies (E) were calculated from the given slopes, according to the equation: E = 10^(-1/slope) ^- 1 × 100 [10], where E = 100 corresponds to 100% efficiency [12]. PCR reactions where the amplicon doubles at every cycle have an optimal efficiency of 100% compared to reactions where no amplification occurs and efficiency is 0%.

To measure relative efficiency, amplifications were performed on the same diluted samples, using primers for the reference (*Pf*-β-*actin1*) and the target genes. The average Ct was calculated for both reference and target genes and the ΔCt (Ct, target gene – Ct, *Pf*-β-*actin1*) was determined. Plots of the log DNA dilution versus ΔCt were made. If the absolute value of the slope was close to zero (m < 0.1), the efficiencies of the target and reference genes were similar, and thus the ΔΔCt calculation could be applied.

## Results and discussion

### Reaction efficiencies and validation of the assay

**Figure 1:**
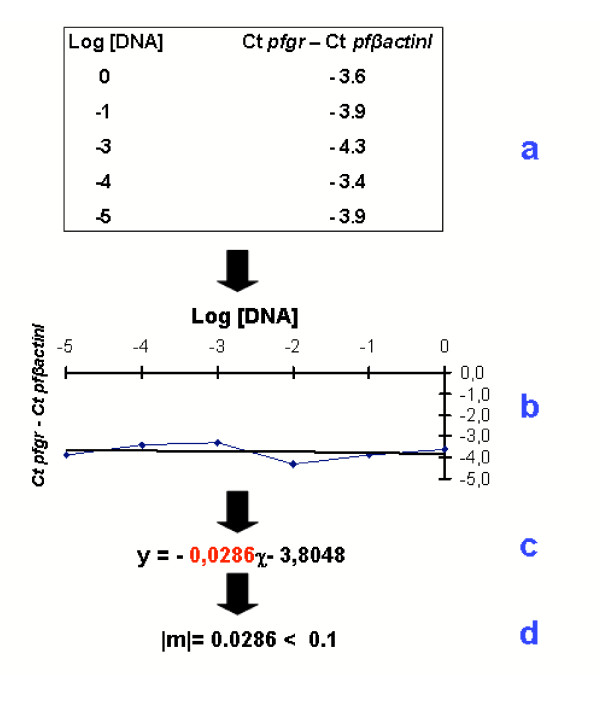
**Calculation of relative efficiency of the *P. falciparum *glutathione reductase gene (*Pfgr*)**. The average Ct at each dilution was calculated for both *Pf*-β-*actin1 *and the glutathione reductase gene, *Pfgr*, and Ct, *Pfgr*-Ct, *P*-β-*actin1 *was determined (a). Plots of the log DNA dilution versus ΔCt were made (b) and a slope was calculated from this (c). The absolute value of this slope (m) was 0.0286 (< 0.1) (d), reflecting optimal relative efficiency.

**Figure 2:**
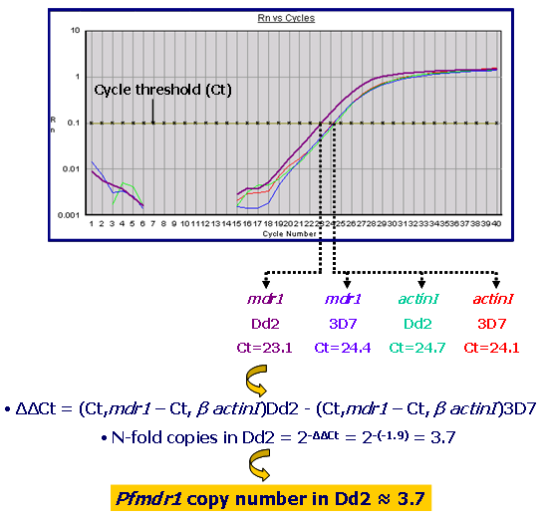
Typical results of estimation of *pfmdr1 *copy number in *P. falciparum *Dd2 relative to 3D7.

Predicted coding sequences obtained for each gene were used to design internal oligonucleotide primers for application in real-time PCR (Figure 2 and Table 3) as described in Materials and Methods section. Using these primers, real-time PCRs were first optimised for each gene by varying both primer and PCR reagent concentrations, in order to obtain optimal efficiencies (data not shown). After a number of rounds of optimisation for each gene individual PCR efficiencies (E) neared 100% in all cases (data not shown). This was also the case for the relative efficiencies between each target gene and *Pf*-β-*actin1*. Therefore, there was no need for efficiency correction. Figure 1 depicts an example assay for the calculation of relative PCR efficiency.

**Table 3:** Estimation of *pfmdr1 *copy number in *P. falciparum *Dd2 relative to 3D7, resulting from the analysis of 20 independent experiments

**Previously reported**	**Observed in present work**			
	Mean	Min.	Max.	SD
4	3.7	3.2	4.5	0.68

To further test the validity of the assays, the copy number of the *Pfmdr1 *gene was compared between the laboratory clones *P. falciparum *3D7 and *P. falciparum *Dd2. The clone 3D7 harbours one copy of the gene while the multidrug-resistant resistant line, Dd2, a derivative of W2mef, contains 4 copies. [3,18]. After ensuring optimal conditions for the *pfmdr1 *assay, the N-fold copy number of Dd2 in comparison to 3D7 was evaluated in twenty independent experiments, as depicted in an example in Figure 2. We note that this system was not directly compared to that reported in references 10 and 11 and in addition, that it would have been more informative to further validate the assay with other *Pfmdr1 *multicopy strains. Nevertheless, the present observations indicated that the assay was robust enough to ensure validation (Table 3).

#### Testing the methods on genomic DNA of natural parasite populations

After optimization of protocols for all different genes the assays were deployed on genomic DNA extracted from confirmed *P. falciparum *infections obtained from eight isolates of the Democratic Republic of São Tomé and Princípe. Parasite densities were determined in Giemsa-stained blood smears and recorded as the number of parasites/μl of blood, assuming an average leukocyte count of 8,000/μl (all smears were examined against 500 leucocytes prior to be declared negative). Parasite densities in these isolates ranged from 2,500 to 500,000 parasites/μl. For measuring the copy number of each gene in each sample, two microlitres of undiluted genomic DNA were used as template for RT-qPCR regardless of parasitaemia. Although it was observed that lower parasite densities caused small shifts of amplification curves to the right (reflecting higher Ct values), that did not influence the outcome of relative kinetic parameters, since data was normalized against the β-*actin1 *gene (data not shown). These experiments showed, however, that there was no amplification in any of the genes among all isolates analysed (Table 4).

**Table 4:** Estimated gene copy numbers in eight *P. falciparum *field-isolates from the DRSTP

**Parasite**	**Copy number relative to *P. falciparum *3D7**								
	*Pfmdr1*	*Pfatp6*	*pftctp*	*pfgpx*	*pfgr*	*pfgst*	*Pfsod1*	*Pftrx1*	*Pftrx2*

Dd2	3.7	1	1	1	1	1	1	1	1
S. Tomé strains	1	1	1	1	1	1	1	1	1

Legend: Eight strains from the islands of São Tomé and Princípe were tested (coded ST060, ST073, ST045, ST013, ST065, ST058 and ST035). For simplification, results are expressed in a single row, since all parasites harboured one copy of the studied genes.

## Conclusion

Protocols were developed to estimate copy numbers of nine putative drug resistance candidate genes in *P. falciparum*. These were initially validated by accurate measure of previously known number of copies of the *pfmdr1 *gene in the drug-sensitive strain *P. falciparum *3D7 and the multi-drug resistant Dd2. Subsequently, protocols were developed for 8 other genes, which were successfully applied to DNA obtained from field isolates of *P. falciparum *collected in the DRSTP. In these particular case, however, all isolates harboured a single copy of the genes studied (Table 4).

The methodology was shown to be sensitive and specific allowing copy number estimations using template DNA extracted with chelex resin from filter paper blood spots, independent of sample parasitaemia. Since all Standard Operating Procedure were designed to function under similar PCR temperatures and reagent concentrations, multiple genes may be analysed in a 96-well optical plate, significantly increasing throughput. The method by-passes the need of gene-specific probes since it relies on SYBR Green I detection and can, thus, be adapted to any given gene provided optimal experimental conditions are assured. As long as primers are properly constructed, most genes are easily and reliably detected with the less cumbersome and less expensive SYBR green I detection method.

Since the methodology relies on relative quantification, it by-passes the need to measure DNA concentration in any given sample. Although a quantification of target genes in genomic DNA is reported, these methods should be equally useful to measure gene expression using cDNA as template, since all primers are located in exons, although it is advisable that PCR efficiencies are calculated and optimized if required, prior to such experiments.

For all reasons cited above, these protocols may be a highly useful tool for high throughput large scale epidemiological assays or laboratory studies.

## Authors' contributions

IDF carried out most of the experimental procedures and contributed for the elaboration of the manuscript. VEdR and PC conceived the study, participated in its design and co-ordination and were involved in phases of the experimental work.
